# COVID-19 and Parkinsonism: A Critical Appraisal

**DOI:** 10.3390/biom12070970

**Published:** 2022-07-11

**Authors:** Francesco Cavallieri, Valentina Fioravanti, Francesco Bove, Eleonora Del Prete, Sara Meoni, Sara Grisanti, Marialuisa Zedde, Rosario Pascarella, Elena Moro, Franco Valzania

**Affiliations:** 1 Neurology Unit, Neuromotor and Rehabilitation Department, Azienda USL-IRCCS di Reggio Emilia, Via Amendola 2, 42124 Reggio Emilia, Italy; francesco.cavallieri@ausl.re.it (F.C.); valentina.fioravanti@ausl.re.it (V.F.); or marialuisa.zedde@gmail.com (M.Z.); 2 Neurology Unit, Fondazione Policlinico Universitario A. Gemelli IRCCS, 00168 Rome, Italy; francescobove86@gmail.com; 3 Neurology Unit, Department of Clinical and Experimental Medicine, University of Pisa, 56126 Pisa, Italy; ele.delprete86@gmail.com; 4 Movement Disorders Unit, Division of Neurology, Grenoble Alpes University, CHU Grenoble Alpes, 38700 Grenoble, France; smeoni@chu-grenoble.fr (S.M.); elenamfmoro@gmail.com (E.M.); 5 Clinical and Experimental Medicine PhD Program, University of Modena and Reggio Emilia, 41121 Modena, Italy; grisanti.sara@gmail.com; 6 Neuroradiology Unit, AUSL-IRCCS di Reggio Emilia, Via Amendola 2, 42124 Reggio Emilia, Italy; rosario.pascarella@ausl.re.it

**Keywords:** COVID-19, extrapyramidal, parkinsonism, Parkinson’s Disease, SARS-CoV-2

## Abstract

A few cases of parkinsonism linked to COVID-19 infection have been reported so far, raising the possibility of a post-viral parkinsonian syndrome. The objective of this review is to summarize the clinical, biological, and neuroimaging features of published cases describing COVID-19-related parkinsonism and to discuss the possible pathophysiological mechanisms. A comprehensive literature search was performed using NCBI’s PubMed database and standardized search terms. Thirteen cases of COVID-19-related parkinsonism were included (7 males; mean age: 51 years ± 14.51, range 31–73). Patients were classified based on the possible mechanisms of post-COVID-19 parkinsonism: extensive inflammation or hypoxic brain injury within the context of encephalopathy (n = 5); unmasking of underlying still non-symptomatic Parkinson’s Disease (PD) (n = 5), and structural and functional basal ganglia damage (n = 3). The various clinical scenarios show different outcomes and responses to dopaminergic treatment. Different mechanisms may play a role, including vascular damage, neuroinflammation, SARS-CoV-2 neuroinvasive potential, and the impact of SARS-CoV-2 on α-synuclein. Our results confirm that the appearance of parkinsonism during or immediately after COVID-19 infection represents a very rare event. Future long-term observational studies are needed to evaluate the possible role of SARS-CoV-2 infection as a trigger for the development of PD in the long term.

## 1. Introduction

Coronavirus disease 2019 (COVID-19), due to severe acute respiratory syndrome coronavirus 2 (SARS-CoV-2) infection, has been associated with many concomitant neurological manifestations, including headache, myalgia, anosmia, and ageusia, as well as neurological syndromes such as encephalopathy, stroke, and coma, among others [1,2,3,4,5]. Between rarer neurological manifestations of COVID-19 infection, movement disorders are increasingly being described, not only among hospitalized patients, but also in milder cases [5,6,7]. Notably, myoclonus and ataxia are the most frequently identified movement disorders in COVID-19 infection [6]. A few cases of parkinsonism linked to COVID-19 infection have been reported so far, raising the possibility of a post-viral parkinsonian syndrome [8,9,10,11,12]. This hypothesis has been supported by: the well-known ability of coronaviruses to enter the central nervous system (CNS) through the olfactory system with subsequent neuronal death [10,11,12]; the presence of higher levels of antibodies against coronaviruses in the cerebrospinal fluid (CSF) of PD patients compared to healthy controls suggesting a possible involvement of viral infection in the pathogenesis of PD [9,10,11,12]; the negative immediate and lasting effects of a COVID-19-related cytokine storm on the CNS with brain cell damage [11], and the possible hypoxic or vascular damage to the basal ganglia caused by COVID-19 infection [9,10,11,12]. The objective of this review is to summarize the clinical–instrumental characteristics of COVID-19-related parkinsonism cases reported in the literature so far and to discuss the possible pathophysiological implications of COVID-19-related parkinsonism.

## 2. Materials and Methods

This systematic review was conducted in accordance with the PRISMA guidelines. A comprehensive literature search was performed using NCBI’s PubMed database and standardized search terms including “Parkinson disease”, “movement disorders” “parkinsonian disorders”, “movement disorders”, “parkinsonian” “parkinsonism”, “extrapyramidal”, “paralysis agitans” and “COVID-19”, “SARS-CoV-2”, and “coronavirus” (Supplementary file).

The search and validity assessments were performed by two independent reviewers (FC and SG). Any disagreement was resolved by consensus with a senior author (FV). Furthermore, the reference lists of all included articles were screened for additional publications. Only articles published in English were included. For the review, we only selected articles reporting cases of COVID-19-related parkinsonism with a detailed clinical and instrumental description. The presence of parkinsonism was classified based on the MDS criteria [13]. We did not include articles reporting aggregated data or cases of COVID-19 infection in PD patients. Furthermore, only patients with a confirmed diagnosis of COVID-19 infection by nasopharyngeal swab were included. Articles that did not report a detailed clinical description of the cases were excluded. Duplicated reports were not included. Relevant qualitative and quantitative data were extracted by two authors (MZ and VF) and were reviewed by a senior researcher (FV). The following variables were included in the data extraction form: age, gender, comorbidities, presence of PD prodromal symptoms, COVID-19 diagnosis (suspected, probable, confirmed), COVID-19 symptoms and severity (mild, moderate, severe, critical) based on the WHO criteria [14], COVID-19 treatment, description of parkinsonian features, genetic testing, additional neurologic manifestations, functional imaging of the dopaminergic system, neuroimaging data, cerebrospinal fluid (CSF), and other relevant laboratory findings. Each patient was classified based on the different mechanisms of post-COVID-19 parkinsonism [12]. In particular, four different mechanisms have been suggested: (1) structural and functional basal ganglia damage mainly involving the substantia nigra pars compacta and nigrostriatal dopaminergic projection; (2) extensive inflammation or even hypoxic brain injury within the context of an encephalopathy; (3) unmasking of underlying but still non-symptomatic PD; or (4) the hypothetical possibility that a viral infection might trigger a series of processes that result in the development of PD over the long term in individuals with genetic susceptibility [12].

In particular, considering that it might be difficult to differentiate between the second and third scenarios, only patients who satisfied the criteria for COVID-19-related encephalitis/encephalopathies proposed in a recent individual patient data meta-analysis [15] were included in the “extensive inflammation or hypoxic brain injury within the context of encephalopathy” group. This allowed us to also include in the “unmasking of underlying still non-symptomatic PD” scenario only patients with no clinical or instrumental signs of extensive inflammation of the brain within the context of encephalitis/encephalopathies, particularly confirmed by the absence of brain MRI pathological changes.

A descriptive statistical analysis of the collected data was performed using IBM SPSS Statistics for Windows version 26.0 (IBM, Armonk, NY, USA). Continuous variables were expressed as the mean (±standard deviation [SD]) and median (range), whereas frequency and percentage were calculated for categorical variables.

## 3. Results

**Figure 1:**
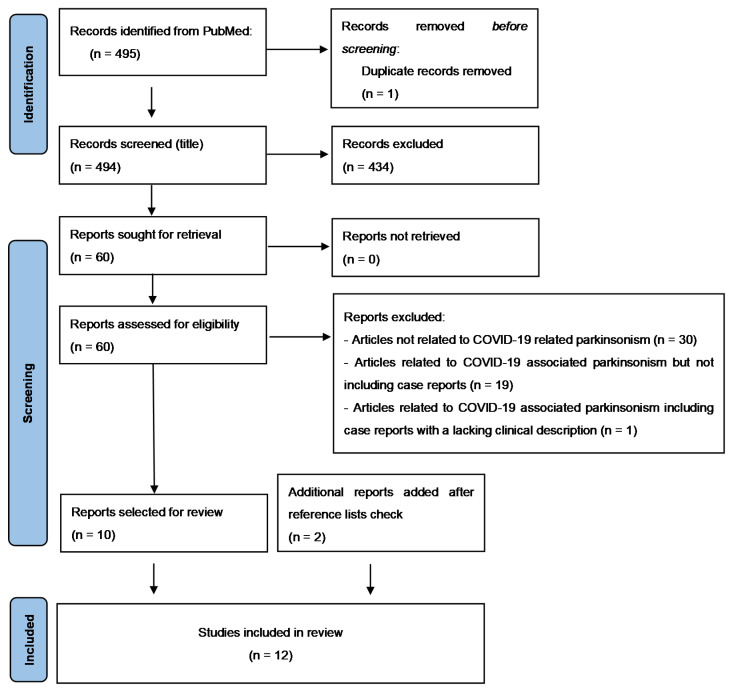
PRISMA flow diagram.

From 1 January 2020 to 1 January 2022, a total of 495 articles were retrieved and one duplicate was removed. Then, 494 articles were identified and reviewed by title and abstract. A total of 430 articles were excluded and, consequently, the remaining 60 full-text articles (case reports or case series: 14, research article: 8, narrative review: 20, systematic review: 3, meta-analysis: 1, editorial/letters to the editor: 14) were assessed. Of them, 50 did not meet the inclusion criteria, while the remaining 10 were included in the systematic review, together with two additional articles that were added through additional sources. Figure 1 illustrates the search process through the PRISMA flow diagram.

### 3.1. Diagnostic Findings

Fourteen cases of COVID-19-related parkinsonism were identified. One patient was not included due to the lack of clinical and neuroimaging data. A detailed description of the remaining 13 patients included in the analysis (eight males; mean age: 52 years ± 14.98, range 31–73) is reported in Table 1. As described above, patients were classified based on the possible mechanisms and scenarios of post-COVID-19 parkinsonism mentioned above [12]. The detailed description of the different subgroups is reported below in Table 1 and Table 2. 

#### 3.1.1. Extensive Inflammation or Hypoxic Brain Injury within the Context of Encephalopathy (n = 5)

In five patients, an inflammatory or hypoxic mechanism within the context of encephalopathy was assumed (three women; mean age: 54.4 years, range 31–73 years). No patients reported prodromal PD symptoms. The severity of the infection was variable between patients: critical (two); severe (two) and moderate (one). The mean interval from COVID-19 infection and the onset of parkinsonism was 35 days (range 0–82). It is interesting to note that, in one case [20], extrapyramidal features were part of the initial clinical manifestations of the infection. In this subgroup, no genetic testing was performed. Clinically, only one patient displayed a resting tremor together with bradykinesia and rigidity, while the remaining four patients presented an akinetic–rigid parkinsonism. In all cases, both sides of the body were affected and the onset was subacute. Only two patients underwent dopamine transporter imaging with (123)I-FP-CIT (DaTSCAN) SPECT and, in all of them, a bilateral, but asymmetric, decreased putaminal uptake was found. Neuroimaging findings were heterogenous in the patents showing: unremarkable brain MRI (three); thalamic and pons T2/FLAIR hyperintensities with hemosiderin deposition (one); and mild cortical atrophy (one). Cerebrospinal fluid analysis was performed in all patients showing only mild elevated protein levels (two); presence of anti-SARS-CoV-2 IgG antibodies and elevated pro-inflammatory cytokines (one); decreased amyloid β42 and increased total Tau protein levels (one); oligoclonal bands (one); and unremarkable findings (one). Four patients were treated with immunomodulant treatments (intravenous [IV] corticosteroids alone or in addition with IV immunoglobulins). In four patients, neuroleptic treatment was used during the course of the infection, mainly due to the appearance of agitation or restless behaviors. Obviously, the neuroleptic treatment could have interfered with the clinical picture; however, in all the reported cases, the authors excluded a possible causal relationship between the onset of parkinsonism and neuroleptic treatment. Dopaminergic drugs were administered in three patients, with a good clinical response in only one of them. Concerning the outcome, parkinsonian symptoms were reversible in three patients, whereas they were still present nine months after discharge in one case. The remaining patient died due to medical complications (infected bedsores and aspiration pneumonia).

#### 3.1.2. Unmasking of Underlying Still Non-Symptomatic PD (n = 5)

In five patients (two women; mean age: 51.2 years, range 35–67 years), COVID-19 infection may have unmasked an underlying but still non-symptomatic PD. In only one case, PD prodromal symptoms (constipation) were reported before the infection. The severity of the infection was mild/moderate in the majority of the cases (four), while in the remaining patient, a severe infection was reported. The mean interval from the infection and onset of parkinsonian symptoms was 48 days (range 5–120 days). genetic assessment was performed in only three patients, revealing a genetic predisposition to PD in two of them. In particular, heterozygous variants in the glucocerebrosidase (GBA) (NM_000157.3:c.1223C > T-p.(Thr408Met); [T369M]) and parkin (PRKN) genes (chr6:162683546–1 62683807NM_004562; exons:3) were found. The majority of patients (four) displayed a parkinsonian resting tremor together with the other parkinsonian features. Two patients showed a unilateral parkinsonism, while in the remaining three patients, both sides of the body were affected. DaTSCAN SPECT was performed and found to be positive in four cases. Brain MRI was normal in four patients, while in the remaining one, only mild, non-specific, chronic cerebrovascular changes were reported. In this subgroup, no neuroleptic drugs were administered before the onset of parkinsonian symptoms. Dopaminergic drugs were administered in four patients (levodopa in two; pramipexole in two) with a good and prolonged clinical response in all of them.

#### 3.1.3. Structural and Functional Basal Ganglia Damage (n = 3)

In three patients (two women; mean age: 47 years, range 35–60), a structural or functional basal ganglia lesion was found. Even in this subgroup, no patients reported PD prodromal symptoms. Two patients developed a critical COVID-19 infection, while the remaining one had a mild infection. The range interval from COVID-19 infection and the onset of parkinsonism ranged between 13 and 41 days. In this subgroup, no genetic testing was performed. Clinically, two cases developed a subacute akinetic–rigid parkinsonian syndrome, while in the remaining one, resting tremor was also reported, in addition to bradykinesia and rigidity. In all cases, the extrapyramidal signs were bilateral. In this subgroup, a DaTSCAN SPECT study was not performed. Brain MRI studies showed basal ganglia lesions in all patients (bilateral edema in the globus pallidus and deep cerebellar nuclei containing small hemorrhagic foci (one); basal ganglia and corona radiata ischemic stroke (one); and bilateral pallidal T2/FLAIR hyperintensities (one). Cerebrospinal fluid analysis was performed only in one patient, and the results were unremarkable. One patient was treated with IV acyclovir followed by IV dexamethasone with subsequent clinical improvement. Even in this subgroup, no neuroleptic drugs were administered. An attempt with dopaminergic drugs (levodopa, range 300 mg to 450 mg daily) was performed in all patients, with a good response in only two of them. The patient who did not respond to levodopa treatment complained of a severe parkinsonian syndrome that was still present one year after the discharge.

## 4. Discussion

### 4.1. Data from Systematic Review

This systematic review summarizes the clinical–instrumental characteristics of the COVID-19-related parkinsonism cases reported so far in the literature. Our results confirm that the appearance of parkinsonism during or immediately after COVID-19 infection represents a very rare event. Obviously, we cannot exclude the possibility that the infection acts as an “initial hit”, leading to a multifactorial process that would eventually end in the long-term development of PD [12]. The notion of a viral cause or trigger of parkinsonism is not new and one of the most famous, although controversial examples is the parkinsonism related to the encephalitis lethargica of von Economo, a viral encephalopathy that developed following the 1918 influenza pandemic [24]. Several viruses, both DNA and RNA, have been associated with acute or chronic parkinsonism [25], starting from the common influenza virus. Several hypotheses have been raised about this association focusing on the direct invasion of the central nervous system by the viruses, causing encephalitis and a milder cytokine response. SARS-CoV2 has many peculiar features promoting the neuroinvasiveness and a strong inflammatory response, so some of the previous hypotheses may be supported. Analyzing the diagnostic findings and the response to dopaminergic treatment in the three subgroups, it can be assumed that the third (structural and functional basal ganglia damage) represents an example of secondary parkinsonism. In this context, COVID-19 infection has indirectly led to structural alterations in the basal ganglia responsible for different signs and symptoms, including parkinsonism [16,18,23]. On the contrary, the second scenario (unmasking of underlying still non-symptomatic PD) might be more intriguing and more interesting. Indeed, in this setting, if we consider COVID-19 infection as a second hit that has unmasked PD in predisposed patients [8,9,21], it would appear extremely appealing to investigate why these subjects were predisposed to developing PD. If this predisposition has a genetic basis, it would be interesting to analyze these subjects for the presence of variants in the main genes involved in the genetic form of PD [26,27]. This is also in view of the possible disease-modifying treatments that are currently studied or will be tested in the near future in patients with LRRK2 or GBA PD [28]. Interesting data has also emerged from functional imaging studies. Presynaptic dopaminergic PET/SPECT imaging is usually used in clinical practice to differentiate degenerative parkinsonism from non-degenerative conditions. The [18F]-DOPA PET and DAT imaging with SPECT radiotracers demonstrate nigrostriatal dysfunction typical of PD as a decreased uptake of the radiotracer in the neostriatum with a predominant early deficit in the putamen and, often, an asymmetric distribution [29]. Of the 13 patients included in this review, only seven had dopaminergic functional imaging: six out of 13 had DAT-SPECT imaging and one out of 13 had 18F-FDOPA PET imaging. All patients who performed dopaminergic functional imaging presented with an altered presynaptic dopaminergic tracer binding. In two out of seven patients, there was a unilateral putaminal reduced uptake, while in the remaining five patients, there was a bilateral putaminal involvement, although with asymmetry in three patients. In this patient cohort, when unilateral/asymmetry in the putaminal tracer uptake was found, this was contralateral to the most clinically affected side. In one patient who presented with an abnormal DaTSCAN SPECT, myocardial MIBG-SPECT was also performed and it revealed cardiac autonomic denervation, as happens in PD. In clinical practice, FDG-PET imaging is increasingly used to improve diagnostic accuracy among parkinsonism [30,31]. In PD patients, FDG-PET imaging usually shows increased metabolic activity in the basal ganglia and thalamus, and decreased activity in the premotor and parietal cortex [32]. Among the 13 patients included in this review, four also underwent FDG-PET brain imaging. In two of these cases, both included in the encephalopathy group, a relative hypermetabolism was found in the mesiotemporal cortex, basal ganglia, brainstem, and cerebellum of both hemispheres associated with diffuse cortical hypometabolism with relatively spared metabolism in the sensorimotor cortex. One out of these four patients presented a normal PET imaging associated with an abnormal DaTSCAN SPECT, while the remaining patient presented with subthalamic hypermetabolism on FDG-PET imaging, a finding already described in Sydenham’s chorea [33], which suggests a possible inflammatory involvement of the basal ganglia.

### 4.2. Possible Pathophysiological Mechanisms of COVID-19 Associated Parkinsonism

Multiple links between viral infections and parkinsonism have been reported in the literature [14]. Several pathophysiological mechanisms underly the possible development of parkinsonism after viral infections, such as induction of neuroinflammation, α-synuclein aggregation, viral neuroinvasivity, blood–brain barrier disruption, and microvascular hypoxic brain injury within the basal ganglia [16]. All of these factors have been postulated to play a role in the pathogenesis of parkinsonism after SARS-CoV-2 infection, and will be discussed in detail in the next paragraphs.

#### 4.2.1. Vascular Damage

The global assessment of the patients summarized in Table 1 shows an heterogeneous cerebral damage pattern, ranging from normal or nonspecific findings on MRI to acute ischemic stroke and an acute encephalopathy finding, including small vessel disease markers already described under the pathophysiological umbrella of “endothelitis” (type 3 hypersensitivity vasculitis) in COVID-19 patients [34,35]. This last mechanism is the best candidate to link inflammatory and microvascular features of cerebral injury seen both on brain MRI and on autopsy studies of COVID-19 patients [35]. It may be responsible for most microhemorrhagic findings in parkinsonian patients of the first and third group, as categorized above. In an autoptic study of six patients [35], four patients had hemorrhages most prominent at the grey and white matter junction of the neocortex, but also in the brainstem, deep grey matter structures, and cerebellum, as some patients in the first and third group. Notably, two patients showed vascular intramural inflammatory infiltrates, consistent with SARS-CoV-2-associated endothelitis. These findings can explain the coexistence of vascular and infectious/inflammatory signs in the reported neuroimaging studies of the patients. The pathological documentation of intramural inflammation in the small vessels of the brain is strictly related to the elevated levels of the SARS-CoV-2 receptor ACE2 in the brain vasculature, and it is associated with sign of severe infection and systemic thrombotic and inflammatory involvement [36,37,38]. In another autoptic series of ten patients [39], all brains had neutrophilic microvascular endothelitis, suggesting that vasculitis with autoimmune features occurred in all patients. This data supports the concept of COVID-19 as a thrombo-inflammatory disease involving both the great and small vessels through different mechanisms. Systemic and cerebral endothelial cells are the main targets of a mixed thrombotic and inflammatory response and the heterogeneous distribution of ACE receptors in the vasculature of the different organs may drive the susceptibility to COVID-19. The high presence of these receptors in the endothelial cells of the brain vessels may be the underlying reason for the increased rates of cerebrovascular disease associated with COVID-19 [40]. In the patients described in the Table 1, it is also possible to find different neuroimaging patterns, suggesting a combination of different mechanisms of damage and a different natural history and response to therapy of the neurological deficit. The description of patient one (Table 1) suggests extrapyramidal features due to encephalitis with basal ganglia damage. In particular, bipallidal T2 hyperintense lesions have been described in a wide range of conditions, from cocaine use [41] to COVID-19 [42,43], and parkinsonism is a key clinical manifestation of this neuroimaging pattern [44]. The neuroradiological picture of patients two and five (Table 1), with a similar clinical scenario, shows a different type of encephalitic involvement in COVID-19, namely acute necrotizing encephalopathy with acute bilateral thalamic lesions, a severe disease prevalent among children in East Asia, outside COVID-19 [45]. This has been described also as severe complication of H1N1 influenza virus infection [46].

#### 4.2.2. Neuroinflammation

Neuroinflammatory alterations have been reported by several neuropathological studies in patients who died from COVID-19 [47]. In particular, microglia and astrocyte activation, neuronophagy, and perivascular and parenchymal T-lymphocyte infiltration were consistently found in the brains of COVID-19 patients [48]. These findings are possibly due to multiple pro-inflammatory mechanisms induced by SARS-CoV-2 infection: viral invasion of the SNc, which promotes local inflammation [49]; systemic inflammation with secondary involvement of the SNc [50]; and disseminated coagulopathy, with cerebral damage and subsequent inflammation [51]. Of note, the small number of autoptic studies conducted in COVID-19 patients so far did not seem to highlight specific neuropathological features, but rather unspecific findings of neuroinflammation, such as the histopathological appearance of other forms of CNS inflammatory disorders [48]. Neuroinflammation plays a main pathophysiological role, not only in the development of post-infective parkinsonism, but also in PD [52]. Experimental and neuropathological studies support the role of inflammation in the propagation and spread of α-synuclein, from the early stages of the disease [53,54]. Moreover, activated T cells and microglia are largely involved in neurodegenerative processes, inducing mitochondrial dysfunction and oxidative stress [55], and directly damaging neurons, astrocytes, and vascular cell types [56]. Systemic inflammation has also been related to an increased risk of developing PD [57], and it may exacerbate ongoing neurodegeneration in PD patient and animal models [58].

Since SARS-CoV-2 is able to induce a marked systemic pro-inflammatory response and central inflammatory alterations, many authors have postulated an increased risk of PD after COVID-19 [11,59,60,61]. Long-term monitoring for neurological sequelae and larger data from autoptic brain examinations will probably clarify the exact mechanisms of post-COVID-19 parkinsonism pathophysiology and the possible impact of SARS-CoV-2 in the risk of developing PD.

#### 4.2.3. SARS-CoV-2 Neuroinvasivity

The exacerbation of neurological symptoms and the manifestation of PD and other neurological disorders in COVID-19 patients highlight the strong impact of SARS-CoV-2 on both the central (CNS) and peripheral nervous systems. A neuroinvasive predisposition of the human SARS-CoV-2 has been suggested [62], similar to other coronaviruses, including SARS-CoV-1, MERS-CoV, HcoV229E, and HcoV-OC43 [62,63] that are associated with several acute brain disorders. The neurotropism of SARS-CoV-2 has been recently examined in transgenic mice expressing human angiotensin-converting enzyme 2 (ACE2) [64], supporting the neuroreplicative potential and lethal consequences of SARS-CoV-2 CNS infection in mice. Similar to other coronaviruses, SARS-CoV-2, as SARS-CoVs, mainly uses the same ACE2 receptor to enter the target cells [65,66]. The ACE2 receptor is expressed in human airway epithelia, lung parenchyma, vascular endothelia, kidney cells, and small intestine cells [67]. Nuclear expression of ACE2 was also found in the brain, in neurons, astrocytes, oligodendrocytes, and endothelial cells, in the human middle temporal gyrus and posterior cingulate cortex [68,69]. Moreover, ACE2 receptors are also expressed in the cardiorespiratory centers in the brain stem, the cerebral cortex, the posterior hypothalamic area, as well as the striatum and dopamine neurons of the substantia nigra [70,71]. The presence of ACE2 receptors in the striatum and substantia nigra, as well as its co-expression with dopamine decarboxylase [72], an enzyme converting L-dopa to dopamine, supports the involvement of SARS-CoV-2 in the pathophysiology of PD induced by viral infection. The neurotropism of SARS CoV-2 was also reported when the virus was found in the CSF and/or in the brain tissue of infected patients, [73,74]. Indeed, while in some COVID-19 patients with encephalitis or demyelinating disease, reverse transcription polymerase chain reaction (RT-PCR) showed the presence of SARS-CoV-2 in CSF samples [75,76], other studies failed to detect the virus in the CSF [77,78,79]. A recent systematic literature review [80] on neuropathological studies in COVID-19, including 438 patients, highlighted the pathogenic relevance of the brain inflammatory reaction and hypoxic-ischemic damage, rather than neuronal viral load, suggesting that different pathogenic mechanisms may coexist in the genesis of COVID-19-related neurological complications. Concerning the entry of SARS-CoV-2 into the brain, several studies suggest that the virus might infiltrate the CNS by the olfactory nerve and/or blood. Preclinical studies, using mouse models expressing human ACE2 infected by intranasal inoculation with SARS CoV-2, have reported the presence of the virus in the brain [81,82]. A recent post-mortem study exploring the pathway of entry of SARS-CoV-2 into the brain [83] has reported the presence of the SARS-CoV-2 S protein and SARS-CoV-2 RNA in the olfactory mucosa and brain areas receiving olfactory tract projections. This study suggests that SARS-CoV-2 neuroinvasion occurs via axonal transport, thus explaining the neurological symptoms in some COVID-19 patients. Interestingly, this study has also shown the presence of SARS-CoV-2 RNA in the cerebellum, a brain region without a direct connection with the olfactory mucosa. This result argues that axonal transport is not the only route of viral entry into the brain, and proposes that SARS-CoV-2 in the CNS endothelium might facilitate vascular damage and allow the virus to spread to other brain regions. SARS-CoV-2 infection may be also associated with a disruption of the blood–brain (BBB) barrier. This disruption is due to viral replication in the cerebral microvascular endothelial cells, causing the degradation of tightly bound proteins [84,85]. In addition, post-mortem examination of the brain of a patient with COVID-19 showed that SARS-CoV-2 was present in cerebral microvascular endothelial cells and frontal lobe nerve tissue [77]. Furthermore, in a prospective cross-sectional study of 102 COVID-19 patients who were PCR-positive for SARS-CoV-2, 50% of the patients with severe neurological symptoms had BBB disruption and elevated interleukin levels in the CSF [86]. Accordingly, BBB leakage was also found in 58% of the 31 COVID-19 patients with neurological complications [87]. Taken together, these findings suggest that SARS-CoV-2 can damage and cross the BBB, leading to neurological complications.

#### 4.2.4. SARS-CoV-2 and α-Synuclein

It has been 100 years since the association between PD and viral infection was hypothesized due to post-encephalitic parkinsonism that occurred after the type A H1N1 influenza pandemic [25]. Later, the association between parkinsonism and viral infection also emerged for other viruses, such as the Epstein Barr virus, Japanese encephalitis virus, coxsackievirus, West Nile virus, western equine encephalomyelitis virus, avian flu virus, and human immunodeficiency virus (HIV) [11]. Neurological symptoms in COVID-19 patients have been related to the neuroinvasion and neurotropism, as happened with the SARS-CoV responsible for a SARS outbreak in 2002−2004 [88]. Several pathophysiological mechanisms have been proposed to explain how COVID-19 infection could represent a risk factor for PD. One of the most captivating proposed mechanisms is related to the role of the ACE2 receptors. The interaction between the SARS-CoV-2 spike protein (S) and ACE2 is crucial for cell infection [60]. A high expression of ACE2 receptors is demonstrated in dopaminergic neurons, the principal cells compromised in PD. The penetration of SARS in these cells could harm and worsen neurological symptoms [89]. This high local expression of ACE2 could explain the selective invasion of the substantia nigra (SN) and basal ganglia in these patients. Moreover, as respiratory and intestinal symptoms are the most common presentation of SARS-CoV-2 infection, the role of nasal and intestinal mucosa involvement might be crucial to the risk of PD in these patients. Indeed, in the Braak dual hit hypothesis, used to explain the spread to the CNS of the α-synuclein in PD [90], the pathogen infection which induces the accumulation of misfolded proteins in peripheral neurons represents the first hit. The second hit is represented by a multifactorial predisposition (genetic predisposition/metabolic condition/age etc.) necessary to facilitate the accumulation of α-synuclein [89]. Finally, the retrograde spread of altered α-synuclein through the vagus or olfactory nerve to SN could represent the natural evolution of PD. Interestingly, SARS-CoV-2 N-protein induces the aggregation of α-synuclein in the test tube [90]. In the presence of the SARS-CoV-2 N-protein, fibril formation is faster and it also proceeds in an unusual way. These results corroborate a direct role of the SARS-CoV-2 N-protein in the pathogenesis of parkinsonism. Unfortunately, when α-synuclein was investigated in the serum/CSF of COVID-19 patients with neurological symptoms, no significant differences were found compared with COVID-19 patients without neurological manifestation or healthy controls. Also, no change in the serum α-synuclein concentration before and after the emergence of neurological manifestations was shown in this work [91]. These data show the controversy about α-synuclein upregulation in humans with neurological symptoms of COVID-19. Another possible mechanism involved in PD risk in COVID-19 patients is neuroinflammation. A multi-systemic inflammatory reaction observed in patients with severe SARS-CoV-2 infection has been already described [92]. Recently, there has been growing interest in the role of inflammation in the pathogenesis of neurodegenerative disorders such as PD. Inflammation can trigger α-synuclein misfolding, aggregation, and propagation through the CNS [60]. Moreover, α-synuclein aggregation could, in turn, induce a pro-inflammatory response and cell damage signaling [60]. In COVID-19 patients, several inflammatory pathways are activated; in particular, cytokine release and microglial activation have been described [49,93]. Among cytokines and chemokines, interleukin (IL)-6, IL-1β, and tumor necrosis factor (TNF)-α involvement is shown in the cytokine storm present in severe COVID-19 infection [89]. When microglia are activated in a pro-inflammatory way they could alter the regulation of the neuroendocrine system, renin–angiotensin–aldosterone and tryptophan–kynurenine pathways, and increase the release of proinflammatory cytokines, chemokines, and neurotoxins in stress-sensitive regions [49,94]. In the SN, a remodeling of the microglia during ageing is also present in healthy subjects. When the microglia are activated the “inflammatory” condition expose the SN to be vulnerable to the environmental event, which could contribute to the onset or progression of PD [49,94,95]. Taken together, all these observations support a possible role of COVID-19 infection in promoting neurodegeneration, and in particular PD.

## 5. Conclusions

In summary, the occurrence of parkinsonism after COVID-19 infection appears to be a rare event that could underline different pathophysiological mechanisms, expressing various clinical scenarios, with different outcomes and response to dopaminergic treatment. Besides the appearance of secondary parkinsonism within the context of COVID-19 infection, SARS-CoV-2 may also act as a second hit that unmasks PD in genetically predisposed patients. Future long-term observational studies are needed to evaluate the possible role of SARS-CoV-2 infection as a trigger for the development of PD in later life stages.

## Figures and Tables

**Table 1:** Clinical context of the 13 patients included in the analysis.

Reference	Age	Sex	Comorbidities	COVID-19 Symptoms	COVID-19 Severity	COVID-19 Treatment	Days to Parkinsonian Features Onset after First COVID Symptoms	Genetic Analysis
Ayele et al., 2021 [16]	35	Female	None	Fluctuating mentation, abnormal behavior, fever, visual hallucination	Mild		13	N/A
Ong et al., 2021 [17]	31	Male	None	Fever, cough, shortness of breath	Severe	Oxygen, dexamethasone, favipiravir, subcutaneous low-molecular-weight heparin.	15	N/A
Cavallieri et al., 2021 [8]	67	Male	None	Dyspnea, fever, anosmia, ageusia	Severe	Tocilizumab	120	Heterozygous variant in the GBA gene (NM_000157.3:c.1223C > T-p.(Thr408Met); [T369M]).
Cavallieri et al., 2021 [8]	45	Male	None	Fever, anosmia and ageusia	Mild		90	Heterozygous variant in the PRKN gene (chr6:162683546–1 62683807NM_004562; exons:3)
Fearon et al., 2021 [18]	46	Male	None	Fever, dyspnea, cough, ARDS, acute renal failure, DIC	Critical	ICU admission, Intubation and ventilation, dialysis	N/A	N/A
Tiraboschi et al., 2021 [19]	40	Female	Overweight	Fever, anosmia, fatigue, dyspnea and one syncope	Critical	ICU admission, intubation, and ventilation	82	N/A
Morassi et al., 2021 [20]	70	Female	Hypertension, anxiety–depressive disorder.	Fever, cough, dysgeusia, bilateral pneumonia	Severe	Darunavir, ritonavir, hydroxychloroquine	47	N/A
Morassi et al., 2021 [20]	73	Female	Hypertension, mixed anxiety–depressive disorder.	Fever, unilateral pneumonia	Moderate		0	N/A
Makhoul et al., 2021 [9]	64	Female	N/A	Fever, fatigue, loss of smell	Mild		5	N/A
Cohen et al., 2020 [10]	45	Male	Hypertension, asthma	Dry cough, muscle pain, loss of smell, fatigue, shortness of breath.	Moderate		17	Negative
Faber et al., 2020 [21]	35	Female	N/A	Fever, cough, diarrhea, myalgia, anosmia, hypogeusia	Mild		10	N/A
Mendez-Guerrero et al., 2020 [22]	58	Male	Hypertension, asthma	Cough, fever, nausea, and shortness of breath, ARDS	Critical	Hydroxychloroquine; Lopinavir/ritonavir, tocilizumab; INF-beta	32	N/A
Roy et al., 2020 [23]	60	Male	Hypertension, diabetes, and hypercholesterolemia	Hypoxic respiratory failure, septic shock, ventricular tachycardia, acute renal failure	Critical	Intubation and mechanic ventilation, convalescent plasma, hemodialysis	41	N/A

Abbreviations: ARDS: acute respiratory distress syndrome; GBA: glucocerebrosidase; ICU: intensive care unit, INF: interferon; N/A: not available; PRKN: parkin.

**Table 2:** Neuroimaging findings and treatment of the 13 patients included in the analysis.

Reference	Parkinsonian Features	Side Involved	Functional Imaging	Brain-MRI	CSF Analysis	Dopaminergic Treatment	Other Treatments	Outcome	Possible Mechanisms of Post-COVID-19 Parkinsonism
Ayele et al., 2021 [16]	Right hand resting tremor, bradykinesia, oromandibular dystonia, rigidity, hypomimia, hypophonia	Bilateral	N/A	Symmetrical T2 and FLAIR hyperintense lesions in both pallidal regions	Unremarkable	Levodopa/carbidopa 250/25 mg half tablet 3/day	IV acyclovir, dexamethasone	Improvement	BG damage
Ong et al., 2021 [17]	Reduced eye blinking, mild bilateral upper limb rigidity, slow finger tapping and absence of arm swing	Bilateral	N/A	Symmetrical T2/FLAIR thalamic hyperintensities with hemosiderin deposition and patchy contrast enhancement.	Mildly elevated protein level.	N/A	IV methylprednisolone, trihexyphenidyl	Significant improvement	Inflammation or hypoxic brain injury in encephalopathy
Cavallieri et al., 2021 [8]	Right hand resting tremor, bilateral bradykinesia rigidity, (MDS-UPDRS-III: 12/132).	bilateral	Mild bilateral reduction in presynaptic dopaminergic uptake	Bilateral mild white matter hyperintensities in the centrum semiovale and external capsule	N/A	Levodopa 300 mg/day	N/A	Good outcome	Unmasking non symptomatic PD
Cavallieri et al., 2021 [8]	Mild resting tremor in left leg and left hand bradykinesia (MDS-UPDRS- III: 4/132)	Left	Decreased dopamine transporter density in both putamens	Unremarkable	N/A	Pramipexole 1.05 mg extended release 1/day	N/A	Good outcome	Unmasking non symptomatic PD
Fearon et al., 2021 [18]	Hypophonia, hypomimia, asymmetric rigidity and bradykinesia, freezing of gait, postural instability.	Bilateral	N/A	CT scan/Brain MRI: bilateral edema in the globus pallidus and deep cerebellar nuclei with hemorrhagic foci.	N/A	Levodopa 450 mg/day	N/A	Lack of improvement one year after COVID-19 infection	BG damage
Tiraboschi et al., 2021 [19]	Parkinsonism	Bilateral	N/A	Unremarkable	Positive for anti-SARS-CoV-2 IgG antibodies and elevated pro-inflammatory cytokines.	N/A	Two IVIg cycles	Complete resolution of symptoms	Inflammation or hypoxic brain injury in encephalopathy
Morassi et al., 2021 [20]	Generalized hypertonia, cogwheel rigidity, bradykinesia hypomimia, hypophonia	Bilateral	Bilateral decrease in presynaptic dopamine involving both putamina, more severe on the left side	Slight enlargement of the ventricular system; fronto-parietal and occipital cortical thinning; fronto-temporal increased cortical thickness.	Decreased amyloid β42, increased total Tau protein	Carbidopa/levodopa (100/25 mg qid)	Corticosteroids followed by five days IVIGs 0.4 g/Kg/die	Modest effect of levodopa. 9 months after presentation: mRS = 4.	Inflammation or hypoxic brain injury in encephalopathy
Morassi et al., 2021 [20]	Bilateral hypokinetic-rigid syndrome.	Bilateral	N/A	Unremarkable	Increased protein content and four oligoclonal bands.	Levodopa/carbidopa up to 100/25 mg qid	Corticosteroids, IVIGs	The patient died of medical complications	Inflammation or hypoxic brain injury in encephalopathy
Makhoul et al., 2021 [9]	Rest tremor in her left arm, minimal hypomimia and mild left-sided bradykinesia and rigidity	bilateral	decreased uptake in the right putamen	N/A	N/A	N/A	N/A	N/A	Unmasking non symptomatic PD
Cohen et al., 2020 [10]	Right more than left tremor, bradykinesia, rigidity	Bilateral	Decreased uptake in bilateral putamen more apparent on the left	Normal	Unremarkable	0.375 mg pramipexole extended release, once daily, biperiden 4 mg daily.	N/A	Tremor improvement after biperiden introduction	Unmasking non symptomatic PD
Faber et al., 2020 [21]	Right rigidity, bradykinesia hypophonia, hypomimia, MDS-UPDRS part III: 49.	Bilateral	Decreased left putamen uptake	Unremarkable		Levodopa/benserazide 200/50 mg three times a day	N/A	Improvement after levodopa introduction	Unmasking non symptomatic PD
Mendez-Guerrero et al., 2020 [22]	Right side–dominant hypokinetic-rigid syndrome, with mixed postural and resting tremor.	Bilateral	Decreased uptake in bilateral putamen more apparent on the left	Unremarkable	Unremarkable	Apomorphine test (3 mg)	N/A	Improvement without any specific treatment	Inflammation or hypoxic brain injury in encephalopathy
Roy et al., 2020 [23]	Diffuse hypokinetic rigid syndrome	Bilateral	N/A	Basal ganglia and corona radiata stroke.		Carbidopa–levodopa 100/25 mg three times a day	N/A	The patient was able to discharge to home after 30 days at the acute rehabilitation center.	BG damage

Abbreviations: BG: basal ganglia; MDS-UPDRS: MDS Unified Parkinson’s Disease Rating Scale; N/A: not available; IV: intravenous; IVIGs: intravenous immunoglobulins; PD: Parkinson’s disease; 3.1.1. Extensive inflammation or hypoxic brain injury within the context of encephalopathy (n = 5).

## Data Availability

Not applicable.

## Supplementary Materials

The following supporting information can be downloaded at: https://www.mdpi.com/article/10.3390/biom12070970/s1, Supplementary file. Standardized search terms.
