# Supplementary material for: COVID-19 and Parkinsonism: A Critical Appraisal
Source: Biomolecules. 2022 Jul 11;12(7):970. doi: 10.3390/biom12070970 (PMC9313170; doi:10.3390/biom12070970)
Supplement: Supplementary file 1 [file biomolecules-12-00970-s001.zip › biomolecules-1769679-Supplementary file.pdf]

(("Parkinson Disease"[Mesh] OR "Parkinson Disease, Secondary"[Mesh] OR "Parkinsonian Disorders"[Mesh] OR "Movement Disorders"[Mesh] OR "parkinsonian"[tw] OR parkinsonian*[tw] OR "parkinsonism"[tw] OR parkinsonism*[tw] OR "parkinson disease"[tw] OR "parkinson's disease"[tw] OR "parkinsons disease"[tw] OR "extrapyramidal"[tw] OR "Paralysis Agitans"[tw] OR parkinson*[tw]) AND ("COVID-19"[Mesh] OR "SARS-CoV-2"[Mesh] OR "COVID-19"[tw] OR "Coronavirus"[tw] OR "Sars-Cov-2"[tw]))
